# Substance Misuse History Documentation at Admission to Secure Rehabilitation Ward Audit

**DOI:** 10.1192/bjo.2024.439

**Published:** 2024-08-01

**Authors:** Elizabeth Westhead, Neeti Sud

**Affiliations:** 1 Cumbria Tyne and Wear NHS Foundation Trust, Newcastle upon Tyne, United Kingdom; 2 Cumbria, Northumberland, Tyne and Wear NHS Foundation Trust, Northumberland, United Kingdom

## Abstract

**Aims:**

To audit compliance with electronic admission documentation relating to substance use.

**Methods:**

The initial admission forms in the electronic records of all current patients on the male ward were reviewed (n = 12). The information in core admission document was compared with other substance abuse history information on records.

**Results:**

Seven of the twelve patients were asked about substance misuse during their admission review. 5 patients were not asked. One of these patients had no history of substance use but his alcohol use history was also unclear in other records. 9 patients had at the very least met the ICD–10 criteria of harmful use of alcohol. 11 patients had at the very least met the ICD 10 criteria for harmful use of illegal substances. Two patients had excessive amount of alcohol use to the point of dependence since teenage. Mean age of onset of both substance and alcohol use was 11, with range of 0 to 20. The most commonly misused substances were alcohol and cannabis (11 out of 12 patients). Eight patients had abused drugs other than cannabis. All but one of these then progressed to using other substances. The reasons for using substances and attitudes to substance and alcohol use were not explored in admission assessment in most cases.

**Conclusion:**

Admission assessment to a rehabilitation ward is also an opportunity to screen for any barriers to recovery as well to use brief motivational interviewing intervention if appropriate clinically. There is a need to improve the quality of our admission assessment in relation to substance use history.

Most of our patients had a very early onset of alcohol and substance use, as young as age 8. Apart from one outlier, all had started using substances and alcohol by age 15. This raises concerns regarding missed early prevention and safeguarding opportunities.

